# I struggle watching her diminish: Caregivers experiences of caring for loved ones with Alzheimer’s

**DOI:** 10.1002/alz.090400

**Published:** 2025-01-09

**Authors:** Lucy Wambui Kamau, Edna N Bosire, Karen Blackmon, Chi Udeh‐Momoh, Olivera Nesic‐Taylor, Dilraj Sokhi, Sylvia Mbugua, Vaibhav Narayan, Zul Merali

**Affiliations:** ^1^ Brain and Mind Institute, Aga Khan University, Nairobi Kenya; ^2^ Aga Khan University, Nairobi Kenya; ^3^ Aga Khan University Hospital, Nairobi Kenya; ^4^ Davos Alzheimers Collaborative, Wayne, PA USA

## Abstract

**Background:**

Alzheimer’s disease is a neurodegenerative disease that affects patients’ ability to perform activities of daily living thus requiring assistance from their loved ones. The progressive nature of the disease unravels new and continuous challenges for the caregivers posing a huge burden on caregiving. However, there is little research in Sub‐ Saharan African countries including Kenya, on caregiver’s experiences managing patients with Alzheimer’s disease. We conducted an ethnographic study at the Aga Khan University Hospital (AKUH) to understand caregivers’ experiences and practices caring for patients with Alzheimer’s disease.

**Methods:**

We purposively recruited 30 caregivers who have been managing patients with Alzheimer’s disease from the Neurology clinic at AKUH. We conducted semi‐structured in‐ depth interviews in English or Swahili, which lasted for about 60 minutes to completion. Interviews were audio‐recorded, transcribed, and analyzed thematically with the aid of Nvivo‐12 software.

**Results:**

Key themes identified from data included: (a) Caregiver knowledge and skills in managing patients (b) Caregiving burden (emotional, psychological, physical, financial) (c) dealing with patient’s changing personality, moods, and loss of self‐identity (d) fulfilment and privilege taking care of loved ones (e) navigating through self‐chores and caregiving roles. Overall, most caregivers lacked knowledge and skills for managing patients with Alzheimer’s. Given the limited resources, awareness and support of Alzheimer’s in Kenya, we found that caregivers carried the burden of taking care of their loved ones with some reporting mental health issues related to caregiving burden. In addition, lack of skills and training on how to manage patients’ changing personalities and patients’ loss of identity left many caregivers frustrated and worn out. Despite the challenges, caregivers had a sense of fulfilment taking care of their loved ones.

**Conclusion:**

Caregivers of Alzheimer’s disease in Kenya require support from healthcare providers and other stakeholders in terms of trainings and capacity building skills to enable them to provide optimal care for the patients. They also require psychosocial support to maintain a healthy balance between their daily life activities and those of caregiving.

